# Comparative safety analysis of lacosamide and perampanel in epilepsy management: insights from FAERS database

**DOI:** 10.3389/fphar.2024.1418609

**Published:** 2024-09-19

**Authors:** Chang Ge, Liuyin Jin, Jing-Jing Tian, Na Yang, Jian Xu

**Affiliations:** ^1^ Department of Obstetrics and Gynecology, Fourth Affiliated Hospital, Zhejiang University School of Medicine, Yiwu, China; ^2^ Lishui Second People’s Hospital, Zhejiang, China; ^3^ Department of Sports, Tsinghua University, Beijing, China; ^4^ Guangdong Pharmaceutical University, Guangzhou, China; ^5^ Department of Obstetrics and Gynecology, Women’s Hospital, Zhejiang University School of Medicine, Hangzhou, China

**Keywords:** epilepsy, lacosamide, perampanel, pharmacovigilance, FAERS, adverse events

## Abstract

**Background:**

Epilepsy is a chronic neurological condition requiring effective management with minimal adverse effects. Lacosamide (LCM) and Perampanel (PER), two promising treatments, have distinct profiles that merit comparative analysis to guide clinical decision-making.

**Methods:**

This study utilizes a pharmacovigilance analysis of adverse events reported in the FDA Adverse Event Reporting System database from Q1 2009 to Q3 2023. Employing disproportionality and Bayesian analyses, we assessed and compared the AE signals associated with LCM and PER to elucidate their safety profiles in epilepsy treatment.

**Results:**

The analysis included 12,576 AE reports for LCM and 2,703 for PER, highlighting a higher incidence of psychiatric disorders, including aggression with LCM, and a notable association of PER with psychiatric disorders such as psychotic disorders and dizziness. LCM showed a relatively safe profile during pregnancy, whereas PER’s data suggested caution due to reported cases of suicidal ideation and attempts.

**Conclusion:**

This comprehensive evaluation underscores the importance of understanding the distinct AE profiles of LCM and PER in clinical practice, providing valuable insights for personalized epilepsy management. Future research with rigorous prospective designs is recommended to validate these findings and explore the mechanisms underlying the reported adverse events.

## 1 Introduction

Epilepsy affects over 50 million people globally, making it one of the most common neurological disorders (Kanner and Bicchi, 2022). The disorder is characterized by the occurrence of spontaneous seizures, which are manifestations of excessive and abnormal neural activity in the brain (Thijs et al., 2019). The management of epilepsy primarily relies on anti-seizure drugs (ASDs), which aim to reduce the frequency and severity of these seizures, thus improving the quality of life for those affected (Charalambous et al., 2014). Among the newer therapeutic options, Lacosamide (LCM) and Perampanel (PER) have emerged as significant additions to the pharmacological arsenal against epilepsy (Li et al., 2020; Yamamoto et al., 2022). LCM, by modulating sodium channels, offers a novel approach to stabilizing neuronal activity and has been incorporated into treatment protocols following its FDA approval in 2008 (Harris and Murphy, 2011). PER, distinct in its mechanism, selectively inhibits AMPA-type glutamate receptors, addressing overexcitation in the brain, a key factor in seizure occurrences (Bresnahan et al., 2023).

However, the introduction of new medications brings forth the challenge of understanding their safety profiles comprehensively. While LCM and PER have been heralded for their efficacy, the spectrum of adverse events (AEs) associated with these drugs raises concerns that warrant careful consideration (Zaccara et al., 2013). For LCM, reports have surfaced indicating potential risks such as dizziness, vision disturbances, and even more serious conditions like cardiac and hematologic anomalies (Roberti et al., 2024). PER’s side effects, including behavioral changes and dizziness, have similarly prompted discussions about its suitability for all patient demographics, especially given the drug’s effectiveness across a broad range of seizure types (Rohracher et al., 2016). Such adverse reactions not only affect patient compliance but also pose a dilemma for clinicians striving to balance therapeutic effectiveness with patient safety (Gaitatzis and Sander, 2013).

Addressing this critical gap, our study harnesses the vast repository of the FDA Adverse Event Reporting System (FAERS) database to undertake a detailed examination of the AEs linked to LCM and PER (Liu et al., 2023). This pharmacovigilance analysis aims to distill crucial data on the nature and frequency of AEs, offering a granular view of the safety landscape surrounding these ASDs. By providing a comparative insight into the adverse profiles of LCM and PER, this research endeavors to equip healthcare providers with the knowledge needed to make informed decisions in epilepsy management. Ultimately, our objective is to contribute to the optimization of epilepsy care, ensuring that treatment decisions are informed by a thorough understanding of the benefits and risks associated with these advanced therapeutic options.

## 2 Methods

### 2.1 Study design and data acquisition

This investigation was conducted as a retrospective pharmacovigilance study, systematically analyzing AE reports derived from the FAERS database spanning from Q1 2009 to Q3 2023. The FAERS database, as a pivotal tool for post-marketing surveillance, compiles AE reports submitted by a diverse cohort including healthcare professionals, consumers, and pharmaceutical entities, thereby facilitating a comprehensive monitoring of drug safety in real-world scenarios. Our analytical focus centered on discerning and contrasting the AE signals attributable to LCM and PER, leveraging their divergent pharmacodynamic profiles.

### 2.2 Data extraction and processing

AE reports pertinent to LCM and PER were meticulously extracted utilizing their generic denominations as search keys. Rigorous scrutiny was applied to each report to ascertain its relevance, followed by the elimination of redundant entries to uphold data integrity. Essential data parameters extracted encompassed demographic details (age and gender), AE narratives, outcomes of the events, and the reporter’s affiliation (healthcare professionals or consumers). The R 4.3.2 and Open Vigil were deployed for the extraction and management of data, enabling an efficient sifting through voluminous datasets and the identification of duplicate records with enhanced accuracy.

### 2.3 Adverse event codification

The codification of AEs was aligned with the terminologies prescribed by the Medical Dictionary for Regulatory Activities (MedDRA), version 25.0. MedDRA’s hierarchical structure facilitates the uniform categorization of AE information, thereby ensuring consistency in reporting across various pharmacovigilance studies. AEs were classified according to the primary System Organ Classes (SOCs) and Preferred Terms (PTs), as delineated in MedDRA, to furnish a granular analysis of the AE profiles associated with both drugs.

### 2.4 Statistical methodology

Signals were coded, classified, and located using preferred terms (PT) and System Organ classes (SOCs) in MedDRA26.1 software to analyze specific SOCs and PTSS involved in adverse event signals. Here we included PT reporting counts ≥3 in our subsequent analysis. In this study, we used four methods: Report Odds Ratio (ROR), Proportional Report Ratio (PRR), Bayesian Neural Network (BCPNN), Multinomial Gamma Poisson (MGPS) for signal recognition and disproportionality analysis. The purpose is to use the advantages of each to expand the detection range, verify the results from multiple perspectives, and make reasonable use of the characteristics of different algorithms to detect more comprehensive and reliable safety signals. The combination of multiple algorithms can be cross-validated to reduce false positives, and more potential rare adverse reactions can be detected by adjusting the threshold and variance. ROR is used to identify the disproportionality of drug-event reporting compared to all other events, and a higher ROR indicates that there may be an underlying signal. The PRR measures the ratio of drug reports to all other drug reports in a given event, and a PRR significantly greater than 1 indicates the presence of a signal. BCPNN computes information component (IC) values using Bayesian logic, with a positive IC indicating a strong correlation. MGPS is a Bayesian data mining method that calculates empirical Bayesian geometric average (EBGM) to assess the strength of the association, with higher EBGM indicating the presence of a stronger signal. When the signal conforms to 1. ROR ≥3 and 95%CI (lower limit) > 1; 2. PRR ≥2 and 95%CI (lower limit) > 1; 3. IC025 > 0; 4. When EBGM05 > 2, we considered the adverse reaction to be significant. See Appendix file. docx for detailed algorithms and formulas.

## 3 Results

### 3.1 Descriptive analysis

#### 3.1.1 Comprehensive analysis of adverse event reporting statistics

**FIGURE 1:**
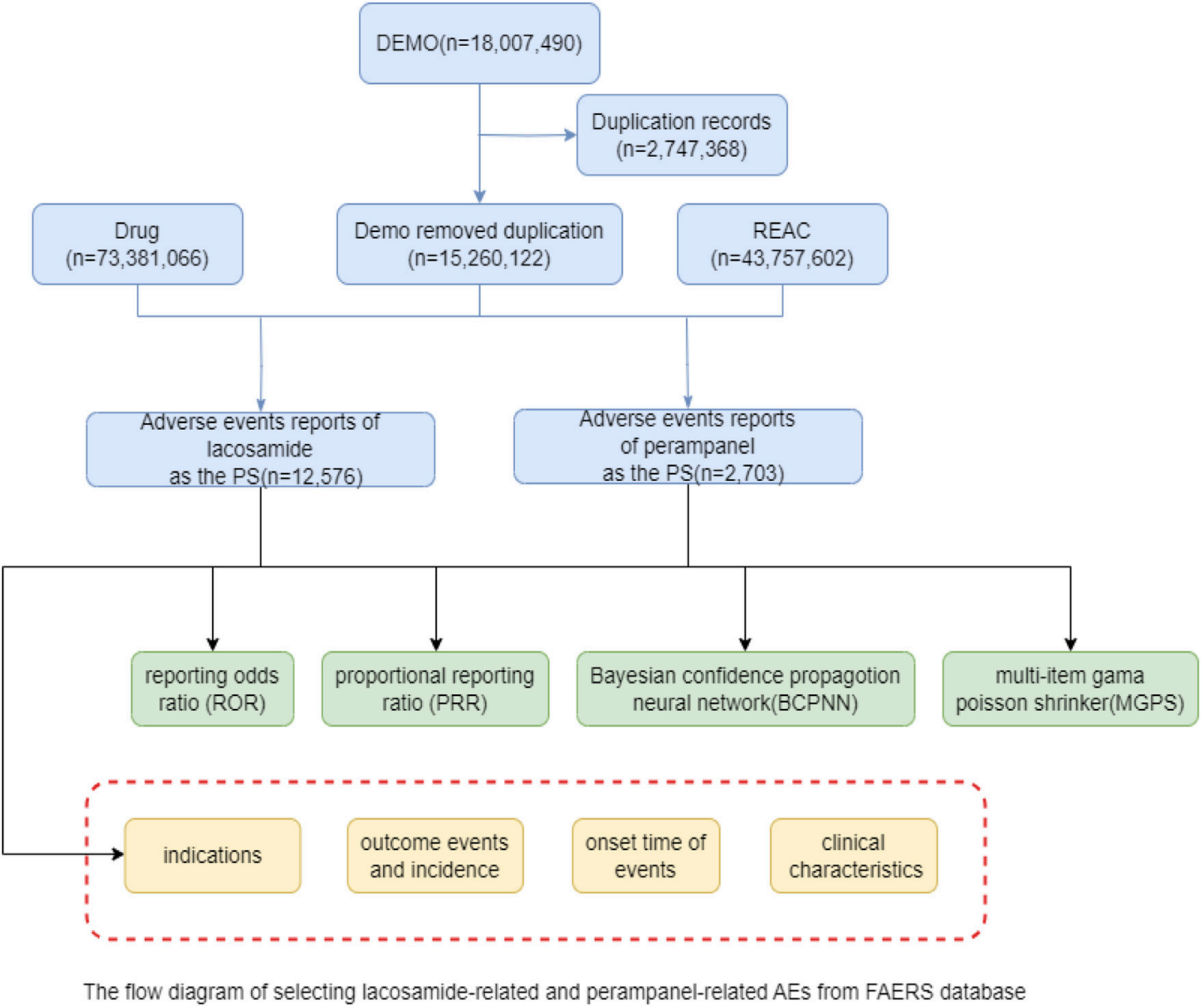
The flow diagram of selecting LCM-related and PER-related AEs from FAERS database.

**FIGURE 2:**
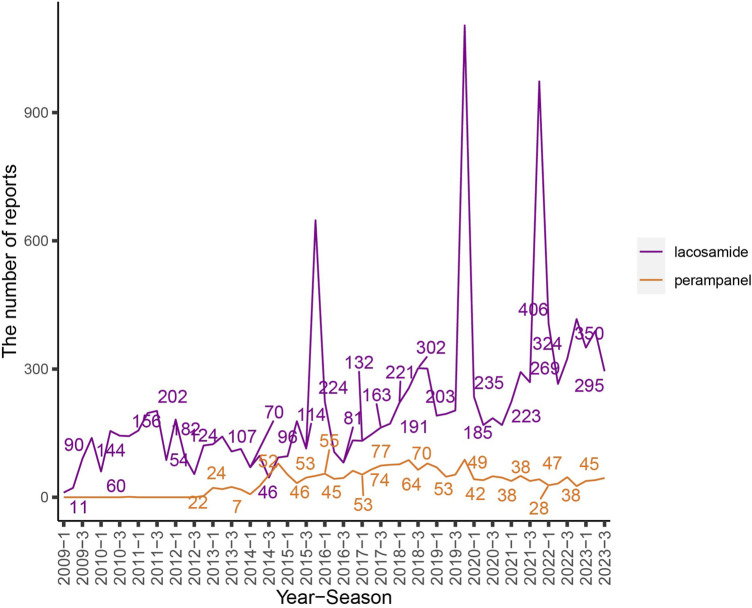
The flow chart and the number of adverse events reported quarterly after the marketing of LCM and PER. Note: The purple line represents the reports of LCM while the orange line represents the reports of PER. *X*-axis shows the timeline when the drug was used, and *Y*-axis displays the number of reports per quarter.

**FIGURE 3:**
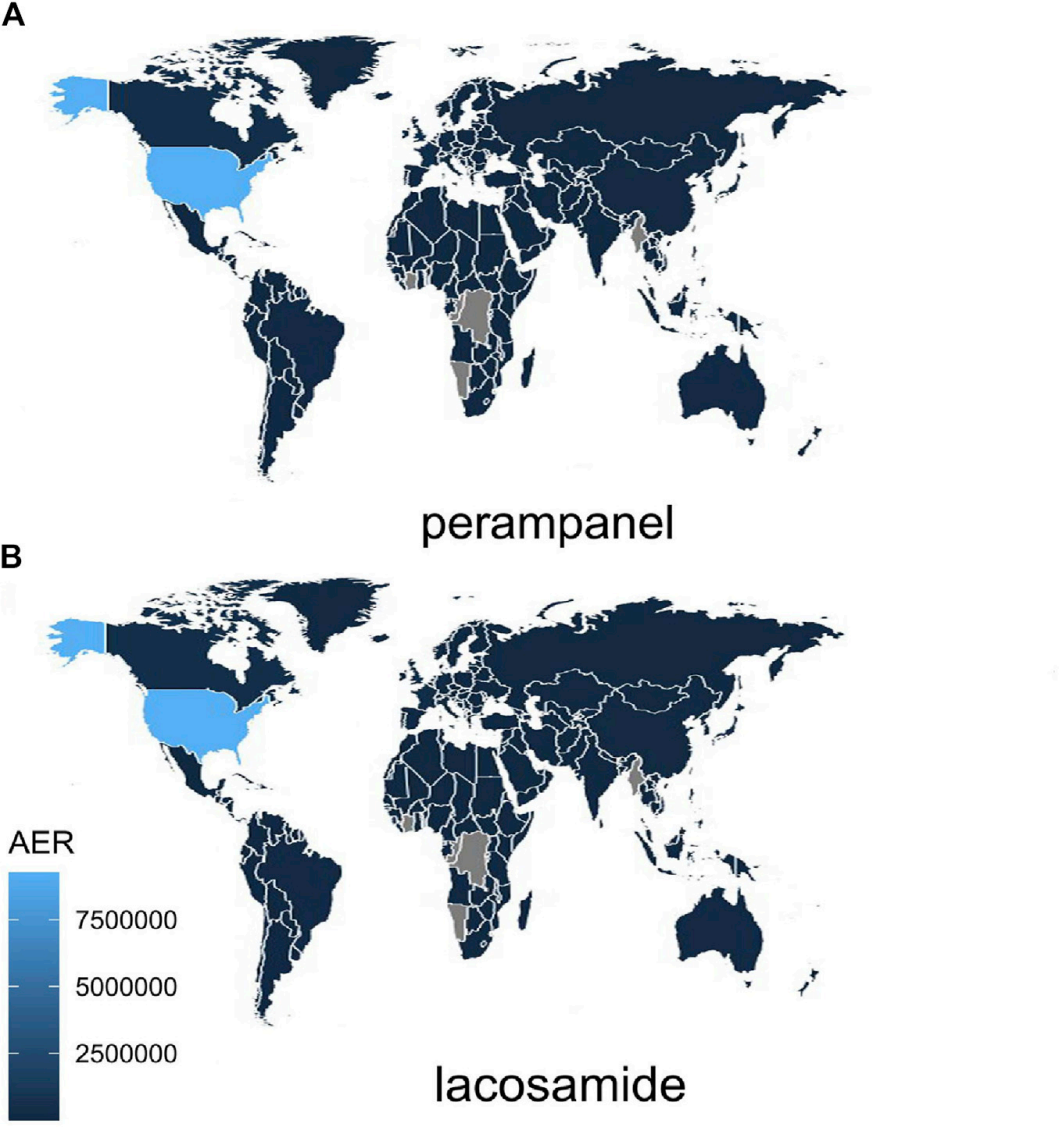
Global Distribution of Adverse Event Reports for LCM and PER note:The varying shades of blue in the map indicate the distribution of adverse event reports for the drug across different countries. Lighter shades correspond to higher report numbers, while grey signifies the absence of AE reports in that particular region. AER, adverse event reports.

The extensive dataset compiled by FAERS is depicted in Figure 1, showcasing a total of 18,007,490 AE reports collected from the first quarter of 2009 to the third quarter of 2023. After cleaning the data, FAERS collected a total of 15,260,122 AE reports, of which 12,576 were related to LCM and 2,703 were related to PER. LCM has an average of 838 AE reports annually. In contrast, PER is a new drug approved for a shorter period of time, whose AE reports do not exist every year, but have been accumulated to 2073 in the past decade, averaging approximately 200 cases per year. Notably, the temporal distribution of these reports reveals a peak in AE reporting for PER in the years 2018 and 2019, with 307 and 259 reports respectively, as detailed in Figure 2 and Table 1. Meanwhile, as shown in Figure 3 and Table 2, the majority of these reports come from the United States, followed by Japan, as well as other countries like Germany and France. This trend suggests a potential increase in the drug’s utilization or possibly an enhanced vigilance in reporting AEs.

**TABLE 1:** Data of reports associated with LCM and PER from Q1 of 2009 to Q3 of 2023.

	LCM	PER
**Number of events**	12576	2073
Year		
2009	262 (2.08)	
2010	502 (3.99)	1 (0.05)
2011	642 (5.10)	
2012	450 (3.58)	3 (0.14)
2013	486 (3.86)	83 (4.00)
2014	306 (2.43)	164 (7.91)
2015	1036 (8.24)	182 (8.78)
2016	544 (4.33)	205 (9.89)
2017	614 (4.88)	268 (12.93)
2018	1079 (8.58)	307 (14.81)
2019	1693 (13.46)	259 (12.49)
2020	758 (6.03)	177 (8.54)
2021	1758 (13.98)	168 (8.10)
2022	1412 (11.23)	133 (6.42)
2023	1034 (8.22)	123 (5.93)

**TABLE 2:** Global distribution of adverse event reports for LCM and PER.

	LCM	PER
Reporter country		
United States	6575 (52.28)	589 (28.41)
Japan	994 (7.90)	506 (24.41)
France	489 (3.89)	175 (8.44)
Germany	658 (5.23)	124 (5.98)
United Kingdom	147 (1.17)	101 (4.87)
Spain	198 (1.57)	86 (4.15)
Italy	251 (2.00)	53 (2.56)
Colombia	276 (2.19)	
Canada	176 (1.40)	
Brazil	152 (1.21)	
Mexico	125 (0.99)	
Australia	54 (0.43)	
Austria	50 (0.40)	
China	50 (0.40)	
other	2381 (18.93)	439 (21.18)

#### 3.1.2 Demographic distribution and reporting sources: an insightful overview

Table 3 presents a detailed demographic breakdown of AE reports related to the treatment of epilepsy with LCM and PER, highlighting a slight predominance of female reporters, constituting approximately 48% of the total reports. Males reported 39.73% and 45.44% of AE reports for LCM and PER, respectively, indicating a nuanced gender distribution in the reporting pattern. The age demographics, primarily concentrated in the 18-45 age group, underline the significance of this cohort in AE reporting, although a considerable fraction of reports did not specify age details. Furthermore, the sources of these AE reports vary significantly between the two drugs. For LCM, consumer reports lead, followed by healthcare professionals, indicating a proactive involvement of patients in reporting AEs. In contrast, for PER, healthcare professionals are the primary reporters, which may reflect a difference in the perceived severity or clinical identification of AEs. This variance in reporting sources is crucial for understanding the pharmacovigilance landscape and is vividly illustrated in Table 3.

**TABLE 3:** Population Information associated with LCM and PER from Q1 of 2004 to Q3 of 2023.

	LCM	PER
Gender		
female	6136 (48.79)	999 (48.19)
male	4996 (39.73)	942 (45.44)
unkown	1444 (11.48)	132 (6.37)
**weight**	68.00 (54.00,83.46)	62.90 (48.00,75.00)
**Age**	48.00 (28.00,65.00)	34.00 (19.00,52.00)
age_yrQ		
<18	925 (7.36)	336 (16.21)
18–45	2378 (18.91)	655 (31.60)
45–65	2013 (16.01)	346 (16.69)
65–75	984 (7.82)	102 (4.92)
≥75	916 (7.28)	67 (3.23)
unknow	5360 (42.62)	567 (27.35)
Reporter		
Physician	4580 (36.42)	1450 (69.95)
Pharmacist	1133 (9.01)	213 (10.27)
Consumer	5696 (45.29)	171 (8.25)
Other health-professional	888 (7.06)	212 (10.23)
route		
oral	4619 (36.73)	2000 (96.48)
other	7311 (58.13)	54 (2.60)
Outcomes		
hospitalization	3590 (30.32)	887 (49.50)
other serious	6258 (52.85)	599 (33.43)
death	1271 (10.73)	126 (7.03)
life threatening	366 (3.09)	110 (6.14)
disability	216 (1.82)	55 (3.07)
congenital anomaly	126 (1.06)	10 (0.56)
required intervention to Prevent Permanent Impairment/Damage	15 (0.13)	5 (0.28)

#### 3.1.3 Delving into drug indications and the temporal dynamics of adverse events

The specific uses of LCM and PER, primarily for epilepsy and seizures, are substantiated by the data in Table 4, showcasing that these conditions account for over three-quarters of all AE reports. The presence of a considerable percentage of reports with unknown indications suggests a gap in documentation or possible off-label use, adding a layer of complexity to drug safety monitoring. The AE profiles for both drugs, as elaborated in Table 5, reveal distinct patterns of side effects. LCM’s AEs are led by overdose (612 case reports), somnolence (405 case reports), and intentional product misuse (291 case reports), indicating specific areas of concern. For PER, aggression (297 case reports), dizziness (152 case reports), and irritability (126 case reports) stand out, suggesting a different set of challenges for patients and healthcare providers. These detailed statistics, presented in Table 5, are pivotal for understanding the risk profiles of these medications. The Time-to-Onset (TTO) analysis, depicted in Table 6, provides an invaluable perspective on the temporal distribution of AEs. LCM exhibits a higher proportion of immediate adverse reactions within 7 days of initiation, whereas PER shows a tendency towards longer-term effects, evident in the significantly higher reports of AEs occurring after 60 days. This temporal aspect of AE reporting, as detailed in Table 6, offers crucial insights into the onset patterns of adverse reactions, enabling more informed clinical decisions and patient management strategies.

**TABLE 4:** Top ten indications in adverse events reports of LCM and PER.

Indications	LCM	n (%)	PER	n (%)
1	seizures	9112 (77.94)	seizures	1576 (76.41)
2	product used for unknown indication	2290 (19.58)	product used for unknown indication	382 (18.52)
3	trigeminalneuralgia	30 (0.26)	syndrome	25 (1.21)
4	syndrome	24 (0.21)	foetal exposure during pregnancy	17 (0.82)
5	migraine	13 (0.11)	accidental exposure to product by child	9 (0.44)
6	neuralgia	9 (0.08)	amyotrophic lateral sclerosis	9 (0.44)
7	pain	8 (0.07)	essential tremor	3 (0.15)
8	prophylaxis	8 (0.07)	infantile spasms	3 (0.15)
9	neuropathy peripheral	8 (0.07)	drop attacks	2 (0.1)
10	off label use	6 (0.05)	dentatorubral-pallidoluysian atrophy	2 (0.1)

**TABLE 5:** Top ten in the number of adverse events reports of LCM and PER.

AE	LCM	n	PER	n
1	overdose	612	aggression	297
2	somnolence	405	dizziness	152
3	intentional product misuse	291	irritability	126
4	memory impairment	277	somnolence	115
5	maternal exposure during pregnancy	245	suicidal ideation	113
6	balance disorder	213	suicide attempt	108
7	bradycardia	194	psychotic disorder	87
8	amnesia	191	anger	78
9	diplopia	185	agitation	76
10	aggression	161	intentional overdose	63

**TABLE 6:** The Time-to-onset (TTO) of adverse events reports of LCM and PER.

	LCM	PER
**tto**	16.00 (0.00,133.00)	34.00 (9.00,124.25)
ttoQ		
<7	957 (17.16)	154 (10.34)
7–28	348 (6.24)	177 (11.89)
28–60	197 (3.53)	138 (9.27)
≥60	801 (14.36)	291 (19.54)
unknown	3275 (58.71)	729 (48.96)

### 3.2 Disproportionality analysis

#### 3.2.1 Analysis of adverse events of LCM and PER

The comprehensive disproportionality analysis of AE reports for LCM and PER, extracted from the FDA’s FAERS) database, reveals significant insights into the safety profiles of these antiepileptic drugs (ASDs). This analysis, grounded in a robust statistical framework, identified 173 and 117 strong signals with an Information Component (IC) of 2 Standard Deviations (SD) ≥ 1.0 for LCM and PER, respectively. As shown in Table 7, these signals, indicative of a statistically significant disproportionality between the observed and expected number of AE reports, highlight potential areas of concern and necessitate a deeper examination of the drugs’ safety profiles. For LCM, the analysis delineates a range of AEs specific to its clinical use. Among the notable findings in the System Organ Classes (SOCs) related to injury, poisoning, and procedural complications, overdose incidents stand out with a χ2 value of 1637.98 and a 95% confidence interval (CI) lower limit of 4.16, reflecting an IC-2SD of 3.69. This is closely followed by intentional product misuse (χ2 = 943.49, 95% CI lower limit = 4.53, IC-2SD = 2.33), and maternal exposure during pregnancy (χ2 = 708.86, 95% CI lower limit = 4.15, IC-2SD = 4.67), underscoring critical areas for clinical vigilance. Additionally, LCM is uniquely associated with memory impairment (χ2 = 404.28, 95% CI lower limit = 2.81, IC-2SD = 1.65) and bradycardia (χ2 = 833.92, 95% CI lower limit = 5.37, IC-2SD = 2.62) within the nervous and cardiac disorder SOCs, respectively. Other specific AEs such as amnesia, diplopia, and head injury further highlight the drug’s diverse impact on patients.

**TABLE 7:** Comparison of single adverse events of LCM and PER from different SOCs.

soc	pt	lacosamide					perampanel				
		Case Reports	ROR (95% CI)	chisq	IC(IC025)	EBGM(EBGM05)	Case Reports	ROR (95% CI)	chisq	IC(IC025)	EBGM(EBGM05)
psychiatric disorders	aggression	161	5.56 (4.76, 6.49)	596.23	2.46 (2.24)	5.52 (4.84)	297	90.7 (80.61, 102.06)	24465.27	6.4 (6.23)	84.29 (76.37)
nervous system disorders	dizziness						152	3.85 (3.28, 4.53)	310.88	1.91 (1.68)	3.76 (3.29)
nervous system disorders	seizure	2466	38.58 (37.01, 40.21)	81584.73	5.13 (5.07)	34.96 (33.77)	138	13.12 (11.07, 15.54)	1498.04	3.67 (3.43)	12.75 (11.07)
psychiatric disorders	irritability	129	3.48 (2.92, 4.13)	225.99	1.79 (1.54)	3.46 (2.99)	126	26.63 (22.3, 31.79)	3016.66	4.69 (4.44)	25.88 (22.31)
nervous system disorders	somnolence	405	3.4 (3.08, 3.75)	676.1	1.75 (1.61)	3.37 (3.1)	115	7.24 (6.02, 8.72)	603.68	2.83 (2.56)	7.09 (6.07)
psychiatric disorders	suicidal ideation						113	17.31 (14.36, 20.87)	1692.64	4.08 (3.81)	16.9 (14.45)
psychiatric disorders	suicide attempt						108	25.29 (20.89, 30.61)	2455.3	4.62 (4.35)	24.67 (21.03)
psychiatric disorders	psychotic disorder						87	42.02 (33.98, 51.98)	3403.86	5.36 (5.06)	41.08 (34.38)
psychiatric disorders	anger						78	30.12 (24.07, 37.69)	2152.39	4.88 (4.56)	29.54 (24.49)
psychiatric disorders	agitation						76	13.98 (11.14, 17.54)	899.78	3.78 (3.46)	13.75 (11.37)
injury, poisoning and procedural complications	intentional overdose						63	12.64 (9.85, 16.21)	665.32	3.64 (3.28)	12.47 (10.13)
general disorders and administration site conditions	gait disturbance						60	3.61 (2.8, 4.65)	111.55	1.84 (1.47)	3.57 (2.89)
nervous system disorders	status epilepticus	361	57.86 (52.04, 64.33)	19083.63	5.78 (5.62)	54.79 (50.14)	55	64.1 (49.08, 83.7)	3350.87	5.97 (5.59)	62.89 (50.31)
general disorders and administration site conditions	drug interaction						54	4.39 (3.35, 5.73)	139.46	2.12 (1.74)	4.35 (3.47)
psychiatric disorders	confusional state						50	3.94 (2.98, 5.21)	108.59	1.97 (1.57)	3.91 (3.1)
psychiatric disorders	abnormal behaviour						48	17.26 (12.99, 22.94)	726.46	4.09 (3.69)	17.07 (13.45)
psychiatric disorders	homicidal ideation						46	200.53 (149.47, 269.04)	8828.92	7.6 (7.18)	193.89 (151.63)
nervous system disorders	ataxia						38	39.93 (28.99, 54.99)	1423.88	5.3 (4.85)	39.43 (30.17)
nervous system disorders	epilepsy	454	26.61 (24.23, 29.22)	10817.33	4.69 (4.55)	25.76 (23.82)	36	15.27 (11, 21.2)	475.48	3.92 (3.45)	15.13 (11.5)
nervous system disorders	balance disorder	213	3.91 (3.41, 4.47)	456.42	1.96 (1.76)	3.88 (3.47)	36	4.94 (3.56, 6.86)	112.22	2.3 (1.83)	4.91 (3.73)
nervous system disorders	altered state of consciousness						35	20.69 (14.83, 28.87)	649.5	4.36 (3.88)	20.5 (15.52)
injury, poisoning and procedural complications	overdose	612	4.51 (4.16, 4.89)	1637.98	2.15 (2.03)	4.44 (4.15)					
nervous system disorders	generalised tonic-clonic seizure	421	43.75 (39.67, 48.24)	16789.8	5.39 (5.25)	41.81 (38.53)					
injury, poisoning and procedural complications	intentional product misuse	291	5.09 (4.53, 5.71)	943.49	2.33 (2.17)	5.04 (4.57)					
nervous system disorders	memory impairment	277	3.16 (2.81, 3.56)	404.28	1.65 (1.48)	3.14 (2.84)					
injury, poisoning and procedural complications	maternal exposure during pregnancy	245	4.71(4.15, 5.34)	708.86	2.22(2.04)	4.67(4.21)					
nervous system disorders	partial seizures	199	70.39 (61, 81.23)	12814.03	6.05 (5.85)	66.32 (58.83)					
cardiac disorders	bradycardia	194	6.18 (5.37, 7.12)	833.92	2.62 (2.41)	6.13 (5.44)					
nervous system disorders	amnesia	191	4.92 (4.26, 5.67)	590.41	2.29 (2.08)	4.88 (4.33)					
eye disorders	diplopia	185	12.53 (10.83, 14.48)	1932.23	3.63 (3.42)	12.35 (10.94)					
nervous system disorders	petit mal epilepsy	166	62.24 (53.24, 72.77)	9482.94	5.88 (5.66)	59.06 (51.82)					
injury, poisoning and procedural complications	head injury	141	7.48 (6.34, 8.83)	784.14	2.89 (2.65)	7.42 (6.46)					
pregnancy, puerperium and perinatal conditions	pregnancy	137	12.94 (10.93, 15.32)	1488.53	3.68 (3.43)	12.77 (11.09)					
general disorders and administration site conditions	multiple-drug resistance	135	86.14 (72.33, 102.59)	10585.65	6.33 (6.08)	80.33 (69.41)					

Conversely, PER exhibits a distinct set of AEs, particularly concentrated within psychiatric disorders. Strong signals were identified for suicidal ideation, suicide attempt, and a spectrum of behavioral disturbances including anger, agitation, confusional state, and homicidal ideation, signifying the drug’s pronounced effects on mental health. The presence of dizziness (χ2 = 310.88, 95% CI lower limit = 3.28, IC-2SD = 1.91) and intentional overdose (χ2 = 665.32, 95% CI lower limit = 9.85, IC-2SD = 3.64) as significant AE signals within the SOC of intentional overdose adds to the safety concerns associated with PER. Moreover, neurological impacts, as evidenced by ataxia (χ2 = 1423.88, 95% CI lower limit = 28.99, IC-2SD = 5.3) and altered state of consciousness (χ2 = 649.5, 95% CI lower limit = 14.83, IC-2SD = 4.36), were exclusively linked to PER, underscoring its potential neurological implications. This analysis not only reinforces the importance of ongoing safety monitoring and evaluation of ASDs but also emphasizes the need for healthcare professionals to be acutely aware of these potential risks when prescribing LCM and PER. Understanding the specific AE profiles of these drugs enables clinicians to devise more informed and individualized treatment plans, enhancing patient safety and therapeutic outcomes in the management of epilepsy.

#### 3.2.2 System disorders analysis of adverse events

LCM’s AEs predominantly affect the nervous system, with a substantial number of reports (n = 9737), showcasing a ROR of 3.97 (95% CI: 3.88-4.07). This is indicative of LCM’s significant neurological effects. Following this, injury, poisoning, and procedural complications also emerge as areas of concern (n = 5630, ROR 1.76, 95% CI: 1.71-1.81), alongside general disorders and administration site conditions (n = 4569, ROR 0.66, 95% CI: 0.64-0.68), psychiatric disorders (n = 3396, ROR 1.7, 95% CI: 1.64-1.76), and gastrointestinal disorders (n = 1624, ROR 0.49, 95% CI: 0.47-0.52). Each of these areas presents a distinct facet of LCM’s impact on patient wellbeing, underscoring the need for comprehensive monitoring and management strategies to mitigate these risks. Conversely, PER exhibits a markedly different AE distribution, most notably in psychiatric disorders, where it presents a significantly higher ROR of 8.76 (95% CI: 8.26-9.3) with 1667 reports. This stark contrast emphasizes the substantial psychiatric risk associated with PER, necessitating vigilant mental health assessments for patients under treatment. Additionally, nervous system disorders (n = 1118, ROR 3.25, 95% CI: 3.04-3.48), general disorders and administration site conditions (n = 427, ROR 0.42, 95% CI: 0.38-0.47), injury, poisoning and procedural complications (n = 419, ROR 0.83, 95% CI: 0.75-0.91), and gastrointestinal disorders (n = 178, ROR 0.4, 95% CI: 0.34-0.46) further delineate PER’s broad spectrum of AEs. This distribution provides essential insights into the drug’s varied effects beyond its primary use, highlighting the complexities of managing its side effects, which can be found in Table 8.

**TABLE 8:** Comparison of system disorders of adverse event signals between LCM and PER.

soc	lacosamide						perampanel					
	Case Reports	ROR (95% CI)	PRR (95% CI)	chisq	IC(IC025)	EBGM(EBGM05)	Case Reports	ROR (95% CI)	PRR (95% CI)	chisq	IC(IC025)	EBGM(EBGM05)
psychiatric disorders	3396	1.7 (1.64, 1.76)	1.63 (1.57, 1.7)	888.09	0.71 (0.66)	1.63 (1.59)	1667	8.76 (8.26, 9.3)	6.07 (5.84, 6.31)	7487.34	2.6 (2.52)	6.07 (5.78)
nervous system disorders	9737	3.97 (3.88, 4.07)	3.15 (3.09, 3.21)	15620.95	1.65 (1.62)	3.14 (3.08)	1118	3.25 (3.04, 3.48)	2.73 (2.57, 2.9)	1338.39	1.45 (1.35)	2.73 (2.58)
congenital, familial and genetic disorders	216	1.92 (1.68, 2.19)	1.91 (1.67, 2.19)	94.21	0.93 (0.74)	1.91 (1.71)	20	1.34 (0.86, 2.08)	1.34 (0.87, 2.06)	1.74	0.42 (-0.2)	1.34 (0.93)
ear and labyrinth disorders	207	1.31 (1.14, 1.5)	1.31 (1.14, 1.5)	14.86	0.38 (0.19)	1.31 (1.16)	29	1.33 (0.92, 1.92)	1.33 (0.92, 1.93)	2.39	0.41 (-0.11)	1.33 (0.98)
pregnancy, puerperium and perinatal conditions	470	3.06 (2.8, 3.36)	3.04 (2.76, 3.35)	642.76	1.6 (1.47)	3.03 (2.81)	21	1.02 (0.67, 1.57)	1.02 (0.66, 1.57)	0.01	0.03 (-0.57)	1.02 (0.72)
hepatobiliary disorders	197	0.61 (0.53, 0.7)	0.61 (0.53, 0.7)	49.56	-0.71 (-0.91)	0.61 (0.54)	38	0.92 (0.67, 1.26)	0.92 (0.67, 1.26)	0.29	-0.13 (-0.58)	0.92(0.7)
injury, poisoning and procedural complications	5630	1.76 (1.71, 1.81)	1.64 (1.61, 1.67)	1553.2	0.71 (0.67)	1.64 (1.6)	419	0.83 (0.75, 0.91)	0.84 (0.76, 0.93)	14.2	-0.25 (-0.39)	0.84 (0.77)
metabolism and nutrition disorders	526	0.67 (0.61, 0.73)	0.67 (0.62, 0.72)	85.26	-0.57 (-0.69)	0.67 (0.63)	84	0.8 (0.65, 1)	0.81 (0.65, 1)	4.01	-0.31 (-0.62)	0.81 (0.67)
eye disorders	720	0.98(0.91, 1.05)	0.98(0.91, 1.06)	0.31	-0.03(-0.14)	0.98(0.92)	67	0.67 (0.53, 0.85)	0.68 (0.54, 0.86)	10.7	-0.57 (-0.91)	0.68 (0.55)
renal and urinary disorders	323	0.46 (0.41, 0.52)	0.47 (0.42, 0.53)	200.75	-1.1 (-1.26)	0.47 (0.43)	50	0.52 (0.39, 0.68)	0.52 (0.4, 0.68)	22.45	-0.94 (-1.34)	0.52 (0.41)
general disorders and administration site conditions	4569	0.66 (0.64, 0.68)	0.7 (0.69, 0.71)	699.68	-0.51 (-0.55)	0.7 (0.69)	427	0.42 (0.38, 0.47)	0.47 (0.43, 0.52)	307.58	-1.08 (-1.22)	0.47 (0.44)
reproductive system and breast disorders	109	0.35 (0.29, 0.42)	0.35 (0.29, 0.43)	130.14	-1.5 (-1.77)	0.35 (0.3)	19	0.47 (0.3, 0.74)	0.47 (0.3, 0.74)	11.29	-1.08 (-1.72)	0.47 (0.32)
investigations	1311	0.59(0.55, 0.62)	0.6(0.57, 0.64)	368.26	-0.73(-0.81)	0.6(0.57)	138	0.45 (0.38, 0.54)	0.47 (0.4, 0.55)	87.6	-1.09 (-1.33)	0.47 (0.41)
skin and subcutaneous tissue disorders	939	0.46(0.43, 0.49)	0.47(0.44, 0.5)	595.44	-1.09(-1.18)	0.47(0.45)	128	0.44 (0.37, 0.53)	0.46 (0.39, 0.55)	86.3	-1.12 (-1.37)	0.46 (0.4)
gastrointestinal disorders	1624	0.49 (0.47, 0.52)	0.52 (0.5, 0.54)	800.12	-0.95 (-1.02)	0.52 (0.5)	178	0.4 (0.34, 0.46)	0.42 (0.37, 0.48)	156.85	-1.25 (-1.47)	0.42 (0.37)
respiratory, thoracic and mediastinal disorders	661	0.37 (0.34, 0.4)	0.38 (0.35, 0.41)	708.39	-1.4 (-1.51)	0.38 (0.36)	97	0.4 (0.33, 0.49)	0.41 (0.34, 0.5)	86.11	-1.28 (-1.57)	0.41 (0.35)
cardiac disorders	1497	1.64 (1.56, 1.73)	1.62 (1.53, 1.72)	360.88	0.69 (0.62)	1.62 (1.55)	46	0.38 (0.29, 0.51)	0.39 (0.29, 0.52)	44.82	-1.36 (-1.77)	0.39 (0.31)
infections and infestations	1086	0.55 (0.51, 0.58)	0.56 (0.53, 0.59)	399.01	-0.84 (-0.92)	0.56 (0.53)	103	0.37 (0.3, 0.45)	0.38 (0.31, 0.46)	108.16	-1.38 (-1.66)	0.38 (0.33)
blood and lymphatic system disorders	330	0.54 (0.48, 0.6)	0.54 (0.49, 0.6)	130.12	-0.88 (-1.04)	0.54 (0.49)	30	0.36 (0.25, 0.52)	0.36 (0.25, 0.51)	33.81	-1.46 (-1.97)	0.36 (0.27)
vascular disorders	387	0.49 (0.44, 0.54)	0.49 (0.44, 0.54)	205.37	-1.02 (-1.16)	0.49 (0.45)	32	0.31 (0.22, 0.43)	0.31 (0.22, 0.44)	50.05	-1.69 (-2.18)	0.31 (0.23)
musculoskeletal and connective tissue disorders	583	0.28 (0.26, 0.31)	0.3 (0.28, 0.32)	1039.02	-1.76 (-1.88)	0.3 (0.28)	67	0.24 (0.19, 0.3)	0.25 (0.2, 0.32)	160.77	-2.01 (-2.35)	0.25 (0.2)
neoplasms benign, malignant and unspecified (incl cysts and polyps)	477	0.45 (0.41, 0.5)	0.46 (0.42, 0.51)	311.47	-1.12 (-1.25)	0.46 (0.43)	24	0.16 (0.11, 0.24)	0.16 (0.11, 0.24)	106.28	-2.62 (-3.18)	0.16 (0.12)
immune system disorders	161	0.39 (0.33, 0.45)	0.39 (0.33, 0.46)	155.59	-1.36 (-1.58)	0.39 (0.34)	9	0.15 (0.08, 0.29)	0.15 (0.08, 0.29)	42.23	-2.7 (-3.59)	0.15 (0.09)
endocrine disorders	45	0.49 (0.37, 0.66)	0.49 (0.37, 0.66)	23.82	-1.03 (-1.44)	0.49 (0.38)						

In the examination of psychiatric disorders of Supplementary Table S1, both LCM and PER exhibited notable signals for aggression. In the realm of nervous system disorders of Supplementary Table S2, a comparative assessment showed that PER had a higher rate of reported drop attacks Contrastingly, in Supplementary Table S3, LCM was found to have a higher risk of causing general disorders and conditions related to administration site.

### 3.3 Time scans of safety signals

**FIGURE 4:**
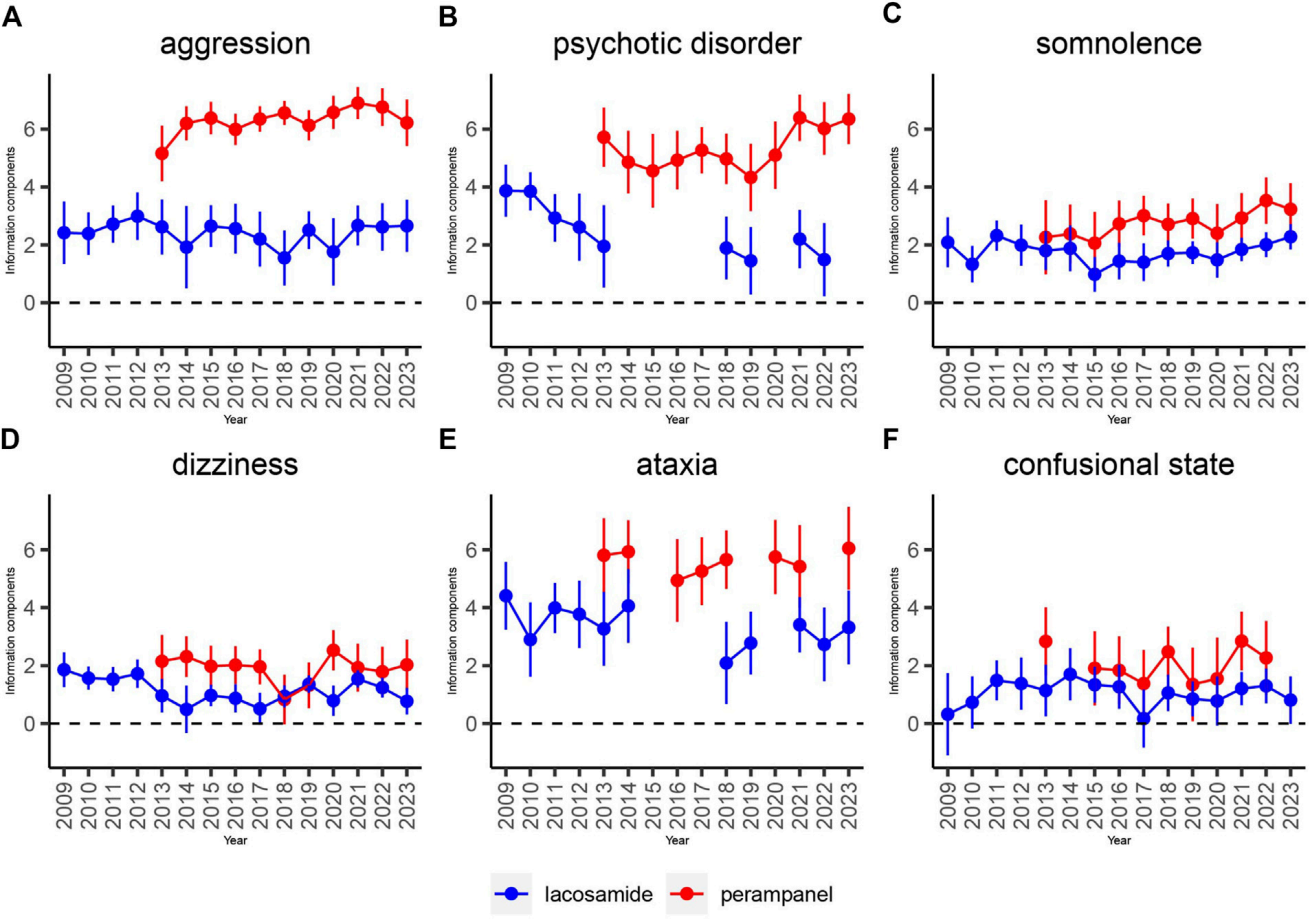
Information component and its 95% credibility interval over time for LCM and PER -associated adverse events. Note: Abbreviations: The blue line represents the reports of LCM while the red line represents the reports of PER; IC, information component; CI, credibility interval. The error bars show the 95% credibility interval (CI) of the information component (IC), when the IC curve is in a steady upward trend and the 95% CI narrowed, the signal is stable and the association is strong. Shrinkage of CI of IC over time with increasing data means that confidence interval gets smaller. Once the value 0 is not included in the CI, a signal is flagged.

To elucidate the evolution of safety signals over time, this study conducted time scans across several keyAEs including aggression, psychotic disorder, somnolence, dizziness, ataxia, and confusional state for both LCM and PER. The graphical analysis revealed a steady or increasing trend in the incidence of these AEs, with the confidence intervals narrowing over time. This pattern indicates a consistent and strong correlation between the occurrence of these AEs and the administration of each drug. Notably, as demonstrated in Figure 4, LCM exhibited a significant correlation with aggression, psychotic disorder, and ataxia, highlighted by an increase in reports and corresponding Information Component (IC) values over time. Somnolence, particularly, saw a rapid increase in IC values to at least 2 within a 6-year period or less, marking the quickest escalation observed in this study.

Conversely, PER’s relationship with these AEs showcased a potentially higher correlation, although the impact of its market release year on this correlation remains to be fully understood. Specifically, dizziness presented a distinct aspect of PER’s AE profile, differing markedly from LCM’s steady trend. This suggests that, despite LCM’s consistent association with the selected AEs, the strength of association for these adverse effects is particularly pronounced with PER.

### 3.4 Comparison of safety signals in four system organ classes

**FIGURE 5:**
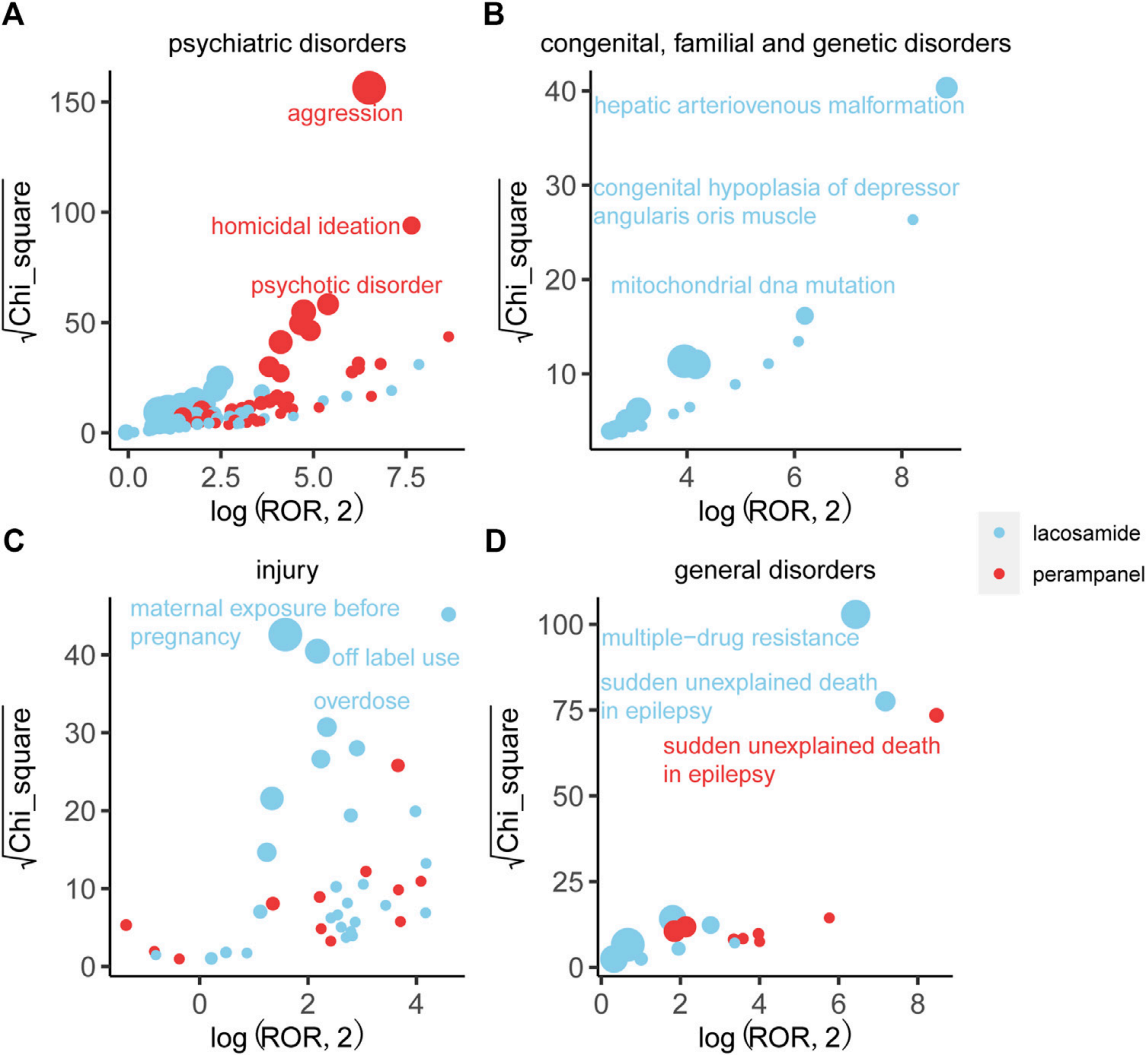
Comparison of four system organ classes safety signals between LCM and PER. Note: **(A)** Blood and lymphatic system disorders; **(B)** Hepatobiliary disorders; **(C)** Respiratory, thoracic and mediastinal disorders; **(D)** Cardiac disorders. Figure 3 respectively shows the mining results of adverse event signals of T-DM1 and T-DXd in four system organ classes. The x-axis is log2ROR, and the y-axis is the square root of the χ2 value. All points in the figure represent the mined adverse reaction signals, and the size of points represents the number of reported adverse reactions. ROR and PRR methods were used to determine the location of each adverse event in the figure. When the position of the point in the graph is higher and further, both algorithms prove that the signal of the adverse event is strong.

An in-depth comparison of AE signals across four major system organ classes unveiled distinct characteristics for each drug, as detailed in Figure 5. PER emerged with aggression in psychiatric disorders as its most prominent signal, underscoring a significant concern in its usage. On the other hand, LCM was closely associated with hepatic arteriovenous malformation within congenital, familial, and genetic disorders, reflecting its unique safety signal based on ROR and Chi-square analyses.

Furthermore, LCM was notably linked to off-label use and overdose within the injury, poisoning, and procedural complications SOC, accumulating 1436 and 612 reports, respectively. These findings suggest a critical area of concern for LCM regarding its administration and usage parameters. Additionally, LCM exhibited the strongest signal for multi-drug resistance across the study, achieving the highest ROR, which highlights significant implications for its clinical management and efficacy. Sudden unexplained death in epilepsy represented another crucial signal for LCM, distinguishing its risk profile from PER. Despite this, the general disorders category for both drugs displayed similar report volumes and minimal differences in ROR and Chi-square, indicating some overlap in their AE spectrums within this SOC.

## 4 Discussion

The escalation in reported AEs for LCM and PER, as observed from 2014 to 2019, underscores the complex landscape of epilepsy management and the vital role of ASDs in this domain (Palleria et al., 2017). This significant increase, highlighted by nearly fivefold growth in annual reports by 2019, reflects not only the expanded indications and enhanced utilization of these medications but also underscores the critical importance of rigorous post-marketing surveillance to safeguard patient health (Abou-Khalil, 2019).

Our comprehensive and systematic analysis, leveraging the vast repository of the FAERS, marks a pivotal stride in understanding the real-world safety profiles of LCM and PER (Hu et al., 2018). By transcending the limitations inherent in clinical trial settings or narrower AE-focused studies, our work provides a panoramic view of the safety issues potentially associated with these drugs. The introduction of innovative time scans scoring in our methodology represents a forward-thinking approach to pharmacovigilance, aiming to preemptively identify and mitigate future safety signals and thus refine the predictive accuracy of AE reporting (Kang et al., 2014).

Epilepsy’s pronounced incidence among young adults and its onset during formative years, influenced by a myriad of factors, is reflected in the age demographics of AE reports analyzed in our study (Manford, 2017). The predominance of reports within the 18-45 age range aligns with the epidemiological understanding of epilepsy, offering a validation of our data’s relevance (Perucca et al., 2018). The slight female preponderance in AE reporting, while not indicative of a significant gender disparity, highlights the necessity for gender-aware management strategies in epilepsy care (Hophing et al., 2022). The disproportionality analysis conducted as part of our investigation reveals nuanced insights into the AE profiles of LCM and PER (Hu et al., 2022). With nervous system disorders and psychiatric disorders emerging as significant concerns, our findings align with and expand upon the safety data available from clinical trials and drug labels (Beghi et al., 2015). This detailed examination not only corroborates known safety profiles but also unveils specific areas requiring vigilant monitoring, such as the heightened incidence of aggression and irritability associated with these ASDs (Kamitaki et al., 2021).

### 4.1 Comparative analysis

Our findings align with and extend the results of previous clinical studies on Lacosamide LCM and PER. Villanueva et al. (Villanueva et al., 2015) reported that LCM has a relatively favorable safety profile with manageable adverse events, which is consistent with our data showing a lower incidence of psychiatric disorders compared to PER. However, our study highlights a significant association of LCM with dizziness and cardiac anomalies, warranting attention during clinical use. On the other hand, Pascarella et al. (Pascarella et al., 2020) and Gasparini et al. (Gasparini et al., 2022) noted that PER is often linked with severe psychiatric disorders including aggression, irritability, and suicidal ideation. Our analysis corroborates these findings and emphasizes the pronounced psychiatric effects of PER, which were observed to have a higher reporting odds ratio and information component values compared to LCM. This comparative insight underscores the need for clinicians to carefully weigh the benefits and risks when selecting these ASDs for epilepsy management.

### 4.2 Age-related analysis

The occurrence of AEs according to patient age reveaed distinct patterns for LCM and PER, which are critical for personalized treatment strategies. Sarkis et al. (Sarkis et al., 2017) documented that older patients treated with LCM exhibited higher incidences of dizziness and ataxia. Our study supports these observations, showing a notable prevalence of these AEs among patients aged 65 and above. In contrast, younger patients, particularly those below 45, reported higher instances of somnolence and memory impairment with LCM.

For PER, Wheless et al. (Wheless et al., 2023) and Pascarella et al. (Pascarella et al., 2023) identified significant age-related differences in AE profiles, with younger patients (under 18) experiencing more behavioral issues and aggression. Our data similarly indicate a higher frequency of psychiatric AEs in younger populations treated with PER. These age-related findings are pivotal for clinicians to consider, as they highlight the differential risk profiles of LCM and PER across various age groups, informing safer and more effective epilepsy management tailored to individual patient needs.

### 4.3 Pregnancy safety profile

The safety of LCM and PER during pregnancy is a crucial consideration, given the potential risks to both the mother and the fetus. Our study found that LCM exhibited a relatively safe profile during pregnancy, with fewer reports of severe AEs compared to PER. Specifically, the data showed 245 AE reports related to maternal exposure during pregnancy for LCM, with an ROR of 4.71 (95% CI: 4.15-5.34) and an IC of 2.22, indicating a strong but manageable risk. These findings are in line with previous studies that suggest LCM can be used with caution during pregnancy due to its lower teratogenic potential.

In contrast, PER’s safety profile during pregnancy raises more concerns. There were 21 reports of AEs related to maternal exposure, with a significant association with severe psychiatric disorders such as suicidal ideation and attempts, as well as aggression. The ROR for PER-related maternal exposure was 1.02 (95% CI: 0.67-1.57), with an IC of 0.03, suggesting a less pronounced but still notable risk profile. These findings align with clinical recommendations to exercise caution when prescribing PER to pregnant women due to its potential adverse effects on mental health.

### 4.4 Potential biases

Our study, while comprehensive, is not without limitations. The reliance on spontaneous reporting to the FAERS database introduces potential biases, including underreporting and variability in report quality. Additionally, the observational nature of our analysis does not allow for causality establishment between drug exposure and AEs, and the absence of a denominator in spontaneous reports limits the ability to calculate precise incidence rates (Kamel et al., 2013). These factors necessitate cautious interpretation of our findings and underscore the need for continuous and multifaceted pharmacovigilance efforts. Despite these limitations, our study significantly contributes to the pharmacovigilance landscape by providing a broad and detailed exploration of the safety profiles of LCM and PER.

### 4.5 Clinical implications

The distinct safety profiles of LCM and PER have significant implications for clinical practice. For instance, the lower incidence of psychiatric AEs with LCM makes it a preferable choice for patients with a history of mental health issues. Moreover, the age-related AE patterns suggest that LCM might be more suitable for older adults, while PER might require careful monitoring in younger patients due to its higher association with behavioral disturbances.

The pregnancy safety profile further influences treatment decisions. LCM’s relatively safer profile during pregnancy could make it a more suitable option for women of childbearing age or those planning to conceive. On the other hand, the heightened psychiatric risks associated with PER necessitate a thorough risk-benefit analysis and close monitoring when prescribed to pregnant patients.

### 4.6 Novelty and future directions

Our study’s novelty lies in its extensive use of the FAERS database to provide a real-world comparative safety analysis of LCM and PER. This approach offers a broader and more detailed view of the AE profiles than clinical trials alone. Future research should focus on prospective studies to validate these findings and explore the underlying mechanisms of the reported AEs. Additionally, integrating genetic and biomarker data could further refine the personalization of ASD therapy, enhancing both efficacy and safety. Our work underscores the dynamic nature of drug safety, highlighting the critical need for ongoing monitoring and evaluation in the post-marketing phase. By elucidating the specific AEs associated with these ASDs and their potential impacts on patient care, we offer valuable insights for healthcare providers (Mesraoua et al., 2020). This knowledge empowers clinicians to make more informed decisions in the management of epilepsy, balancing therapeutic efficacy with patient safety. Furthermore, our findings stress the importance of personalized treatment strategies that consider individual patient factors and potential AE risks. The enhanced understanding of AE profiles provided by our study can guide clinicians in optimizing treatment plans, ultimately improving patient outcomes in epilepsy care.

## 5 In conclusion

Our comprehensive analysis emphasizes the indispensable role of pharmacovigilance in optimizing epilepsy management. As LCM and PER continue to play crucial roles in treating this challenging condition, our study contributes to a deeper understanding of their safety profiles, facilitating informed clinical decision-making and enhancing patient care. Future research, armed with more robust pharmacovigilance methods, will continue to build upon our findings, further advancing the goal of safe and effective epilepsy treatment.

## Data Availability

The original contributions presented in the study are included in the article/Supplementary Material, further inquiries can be directed to the corresponding authors.

## Supplementary material

SUPPLEMENTARY TABLE S1 Comparison of adverse event signals between LCM and PER in psychiatric disorders.

SUPPLEMENTARY TABLE S2 Comparison of adverse event signals between LCM and PER in nervous system disorders.

SUPPLEMENTARY TABLE S3 Comparison of adverse event signals between LCM and PER in general disorders and administration site conditions.
