# Supplementary material for: Comparative safety analysis of lacosamide and perampanel in epilepsy management: insights from FAERS database
Source: Front Pharmacol. 2024 Sep 19;15:1418609. doi: 10.3389/fphar.2024.1418609 (PMC11446851; doi:10.3389/fphar.2024.1418609)
Supplement: Supplementary file 1 [file DataSheet1.PDF]

Methodology of Disproportionality Analysis

	Drug-related ADEs	Non-drug-related ADEs	Total
Drug	a	b	a + b
Non-drug	c	d	c + d
Total	a + c	b + d	N = a + b + c + d

Method	Formula	Threshold
ROR	$\mathrm{ROR} = \frac{\mathrm{a} / \mathrm{c}}{b / d}$	a ⩾ 3<br>ROR ⩾ 3<br>95%CI<br>(lower<br>limit) > 1
	$SE(lnROR) = \sqrt{\frac{1}{\mathrm{a}} + \frac{1}{\mathrm{b}} + \frac{1}{\mathrm{c}} + \frac{1}{\mathrm{d}}}$	
	$95\%CI = \mathrm{e}^{\ln(ROR) \pm 1.96se}$	
PRR	$\mathrm{PRR} = \frac{\mathrm{a} / (\mathrm{a} + \mathrm{b})}{c / (c + d)}$	a ⩾ 3<br>PRR ⩾ 2<br>95%CI<br>(lower<br>limit) > 1
	$SE(lnPRR) = \sqrt{\frac{1}{\mathrm{a}} - \frac{1}{\mathrm{a} + \mathrm{b}} + \frac{1}{\mathrm{c}} - \frac{1}{\mathrm{c} + \mathrm{d}}}$	
	$95\%CI = \mathrm{e}^{\ln(PRR) \pm 1.96se}$	
BCPNN	$\mathrm{IC} = \log_2 \frac{p(x,y)}{p(x)p(y)} = log_2 \frac{a(a+b+c+d)}{(a+b)(a+c)}$	IC025>0
	$\mathrm{E(IC)} = \log_2 \frac{(a+\gamma 11)(a+b+c+d+\alpha)(a+b+c+d+\beta)}{(a+b+c+d+\gamma)(a+b+\alpha 1)(a+c+\beta 1)}$	
	$\mathrm{V(IC)} = \frac{1}{(ln2)^2}[\frac{(a+b+c+d)-a+\gamma-\gamma 11}{(a+\gamma 11)(1+a+b+c+d+\gamma)} + \frac{(a+b+c+d)-(a+b)+a-\alpha 1}{(a+b+\alpha 1)(1+a+b+c+d+\alpha)} + \frac{(a+b+c+d+\alpha)-(a+c)+\beta-\beta 1}{(a+b+\beta 1)(1+a+b+c+d+\beta)}]$	
	$\gamma = \gamma 11 \frac{(a+b+c+d+\alpha)(a+b+c+d+\beta)}{(a+b+\alpha 1)(a+c+\beta 1)}$	
	$\mathrm{IC} - 2\mathrm{SD} = \mathrm{E(IC)} - 2\sqrt{\mathrm{V(IC)}}$	
EBGM	$\mathrm{EBGM} = \frac{a(a+b+c+d)}{(a+c)(a+b)}$	EBGM05>2
	$SE(lnEBGM) = \sqrt{\frac{1}{\mathrm{a}} + \frac{1}{\mathrm{b}} + \frac{1}{\mathrm{c}} + \frac{1}{\mathrm{d}}}$	
	$95\%CI = \mathrm{e}^{\ln(EBGM) \pm 1.96se}$	
